# Draft genomes of three *Sneathia vaginalis* isolates from a patient with bacterial vaginosis

**DOI:** 10.1128/mra.00941-24

**Published:** 2025-02-12

**Authors:** Jonathan J. Panzer, Natalie McGinnis, Jochen Blom, Andrew D. Winters, Jack D. Sobel, Kevin R. Theis

**Affiliations:** 1 Department of Biochemistry, Microbiology, and Immunology, Wayne State University School of Medicine, Detroit, Michigan, USA; 2 Bioinformatics and Systems Biology, Justus-Liebig-University Giessen, Giessen, Germany; 3 Department of Physiology, Wayne State University School of Medicine, Detroit, Michigan, USA; 4 John D. Dingell Veterans Affairs (VA) Medical Center, Detroit, Michigan, USA; 5 Department of Internal Medicine, Wayne State University School of Medicine, Detroit, Michigan, USA; 6 Department of Obstetrics and Gynecology, Wayne State University School of Medicine, Detroit, Michigan, USA; Rochester Institute of Technology, Rochester, New York, USA

**Keywords:** *Sneathia*, bacterial vaginosis

## Abstract

*Sneathia vaginalis,* a fastidious pathogen of the female reproductive tract, is implicated in obstetric and gynecologic pathologies, including spontaneous preterm birth and bacterial vaginosis. Here, we report the successful cultivation and genomic sequencing of three *Sneathia vaginalis* isolates collected via a vaginal swab from a patient with bacterial vaginosis.

## ANNOUNCEMENT

*Sneathia vaginalis* is one of the two currently known *Sneathia* species within the family Leptotrichiaceae, the other being *Sneathia sanguinegens* (1–4). Bacteria of the genus *Sneathia* are known to be associated with human mucosal surfaces, such as the oral cavity (5, 6), gastrointestinal tract (5, 7), and principally the vagina (8–10). Due to their fastidious nature relative to other bacteria in the vaginal microbiome, previous isolation of *Sneathia* spp. has largely been accomplished through anaerobic blood culture of patients with systemic or intra-amniotic infection (1, 2). While this has led to the publication of the genomes of six *Sneathia* isolates, a better understanding of *Sneathia* pathology, especially in the primary context of obstetrics and gynecology, will only be gained through broader cultivation that does not depend on systemic infection for feasible isolation. The initial focus needs to be on the comparative genomics of *Sneathia* inhabiting the vagina, yet only a single such isolate has been characterized to date (9). Considering that *Sneathia vaginalis* is the more common *Sneathia* species in the human vaginal microbiome (9) and that its abundance is increased during episodes of bacterial vaginosis (11, 12), we targeted its isolation through vaginal swabbing of patients with bacterial vaginosis. Here, we report the genomic characterization of three *S*. *vaginalis* isolates from a single patient.

The vaginal swab was collected from a patient at Wayne State University in Detroit, MI, USA. The participating woman provided written informed consent prior to sample collection. After collection, the vaginal swab transport solution (anoxic 5% defibrinated sheep’s blood in PBS) was diluted and spread over chocolate agar (Fisher Scientific, Waltham, MA, USA) prepared in advance with 100 µL of human serum. After 72 hours of incubation at 37°C in an anaerobic chamber, bacterial colonies with morphologies consistent with *Sneathia* spp. were isolated and replated on chocolate agar with human serum. After an additional 72 hours of incubation at 37°C in an anaerobic chamber, DNA extraction on all bacterial growth from restreaked isolates was performed with the DNeasy Powersoil Pro Kit (Qiagen, Germantown, MD, USA). Quantification of DNA extracts was performed with a Qubit 3.0 fluorometer. Library preparation and sequencing were performed by the Research Technology Support Facility Genomics Core at Michigan State University. Libraries were prepared using the Roche KAPA HyperPrep DNA kit (Indianapolis, IN, USA) with KAPA Unique Dual Index adapters following the manufacturer’s recommendations. After quality control and quantification, libraries were normalized and pooled. Sequencing was performed on an Illumina MiSeq (2 × 250-bp reads). Sequencing produced 862,595 pairs of reads, with an average of 277,384 reads per isolate. Raw reads were assembled with strategic k-mer extension for scrupulous assemblies version 2.5.1 (13) and annotated with the Prokaryotic Genome Annotation Pipeline (PGAP) version 2024-04-27.build7426 (14). Default parameters were kept except for the specification of singularity as the docker-compatible executable, *Sneathia vaginalis* as the organism’s name, and the inclusion of the “--ignore-all-errors” flag for the PGAP pipeline to ignore internal N’s.

**Fig 1:**
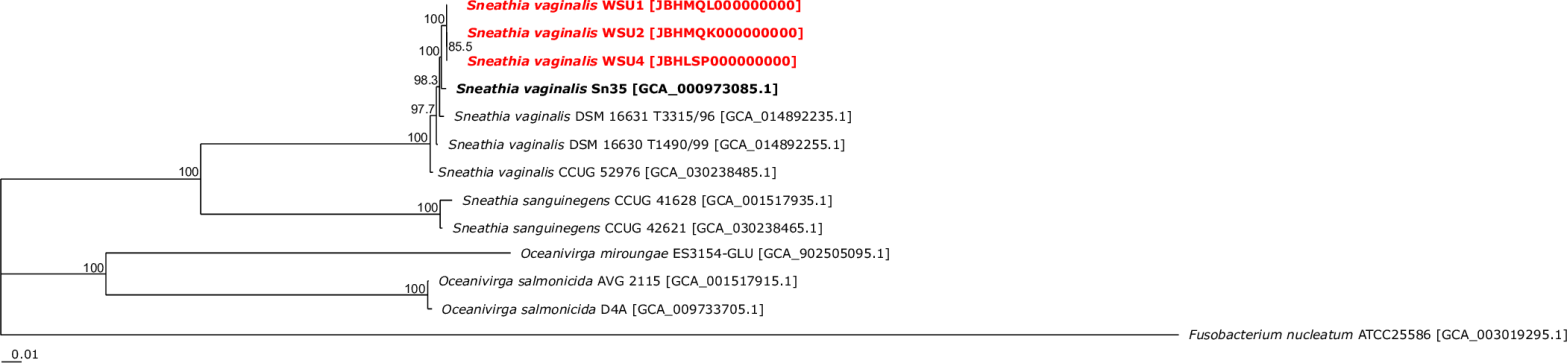
Core genome phylogenetic tree depicting *Sneathia vaginalis* isolates WSU1, WSU2, and WSU4 compared to the six publicly available *Sneathia* spp. genomes excluding metagenome-assembled genomes. All assemblies were annotated with PGAP version 2024-04-27.build7426. Core genes of these genomes were computed in EDGAR version 3.2 by checking for reciprocal best BLAST hits against all other genomes with *Sneathia vaginalis* Sn35 as the reference genome (15). Alignments of each core gene set were generated with MUSCLE and concatenated (16). A phylogenetic tree was constructed with the neighbor-joining algorithm as implemented in the PHYLIP package (17). To verify the tree topology, 1,000 bootstrap iterations were calculated. Branch support was above 85.5% for all branches, with all but three showing 100% support. The core consisted of 433 genes per genome in 13 genomes (5,629 genes in total). The core alignment had 175,990 amino acid residues (2,287,870 residues in total). GenBank accession numbers are given in square brackets. *Sneathia* spp. isolated from vaginal swabs were highlighted in bold. Three genomes from the closely related genus *Oceanivirga* and a *Fusobacterium nucleatum* genome were included for comparison. Bar length is equivalent to 0.01 nucleotide substitutions per site.

All three draft genomes exhibited an average nucleotide identity (ANI) greater than 97% when compared to the four currently published *Sneathia vaginalis* genomes. In contrast, ANI ranged from 77.18% to 77.57% when these draft genomes were compared to the two published *Sneathia sanguinegens* genomes. Together with the phylogeny based on a core gene set of 433 genes (Fig. 1), ANI indicated that these three isolates could confidently be placed within the species *Sneathia vaginalis*. The core genome of *Sneathia vaginalis*, including these three isolates (Table 1), consisted of 998 coding DNA sequences (CDSs) (1,018 CDSs without the new isolates), while the pangenome consisted of 1,887 CDSs (1,798 CDSs without the new isolates). Along with future isolates, these draft genomes will enable the characterization of the core genomic features of *Sneathia* spp. and facilitate elucidation of their genomic variability at the species and strain level.

**TABLE 1:** Genomic features of *S. vaginalis* isolates WSU1, WSU2, and WSU4

Features	*Sneathia vaginalis* isolates		
	WSU1	WSU2	WSU4
Paired-end reads	271,186	277,883	283,083
Genome size (bp)	1,282,629	1,281,601	1,281,879
Genome coverage (×)	84.028	82.776	80.71
GC content (%)	28.31	28.31	28.31
No. of contigs	45	48	52
*N*_50_ (bp)	55,341	51,614	54,865
Protein coding genes	1,206	1,206	1,211
Genome completeness (%)—CheckM	91.92	91.92	91.92
Genome contamination (%)—CheckM	0.56	0.56	0.56
Genome accession numbers	JBHMQL000000000	JBHMQK000000000	JBHLSP000000000
Sequence Read Archive accession numbers	SRR30275402	SRR30275401	SRR30275400

## Data Availability

The annotated draft genomes of *Sneathia vaginalis* isolates WSU1, WSU2, and WSU4 are available at the National Center for Biotechnology Information (NCBI) under BioProject PRJNA1149023 (accession numbers JBHMQL000000000, JBHMQK000000000, and JBHLSP000000000). Raw sequence data from Illumina MiSeq are available at the Sequence Read Archive (accession numbers SRR30275402, SRR30275401, and SRR30275400).
